# Community participation in rural health: a scoping review

**DOI:** 10.1186/1472-6963-13-64

**Published:** 2013-02-18

**Authors:** Amanda Kenny, Nerida Hyett, John Sawtell, Virginia Dickson-Swift, Jane Farmer, Peter O’Meara

**Affiliations:** 1 La Trobe Rural Health School, La Trobe University, P.O Box 199, Bendigo, Victoria, 3550, Australia

**Keywords:** Community participation, Community engagement, Rural health, Health policy, Health reform, Health services

## Abstract

**Background:**

Major health inequities between urban and rural populations have resulted in rural health as a reform priority across a number of countries. However, while there is some commonality between rural areas, there is increasing recognition that a one size fits all approach to rural health is ineffective as it fails to align healthcare with local population need. Community participation is proposed as a strategy to engage communities in developing locally responsive healthcare. Current policy in several countries reflects a desire for meaningful, high level community participation, similar to Arnstein’s definition of citizen power. There is a significant gap in understanding how higher level community participation is best enacted in the rural context. The aim of our study was to identify examples, in the international literature, of higher level community participation in rural healthcare.

**Methods:**

A scoping review was designed to map the existing evidence base on higher level community participation in rural healthcare planning, design, management and evaluation. Key search terms were developed and mapped. Selected databases and internet search engines were used that identified 99 relevant studies.

**Results:**

We identified six articles that most closely demonstrated higher level community participation; Arnstein’s notion of citizen power. While the identified studies reflected key elements for effective higher level participation, little detail was provided about how groups were established and how the community was represented. The need for strong partnerships was reiterated, with some studies identifying the impact of relational interactions and social ties. In all studies, outcomes from community participation were not rigorously measured.

**Conclusions:**

In an environment characterised by increasing interest in community participation in healthcare, greater understanding of the purpose, process and outcomes is a priority for research, policy and practice.

## Background

Rural health is identified as a key priority for health reform across the United States [1-3], Canada [4], the United Kingdom [5,6], Europe [7], Asia [8] and Australia [9-13] due to complex access and equity issues associated with geographic distance, socially determined disadvantage, mal-distribution of health professionals, scant resources and poorer health outcomes across key indicators [1-15]. However, internationally, there is increasing recognition that while rural areas share some commonality, health inequalities vary considerably, requiring locally targeted responses that align with local population health need [4,5,11,13,14]. Accordingly, international policy is increasingly identifying the role of communities in healthcare planning, design, delivery and evaluation to avoid an ineffective ‘one size fits all’ approach [2,4,6,12,16].

In 1978, the World Health Organisation [17] identified the centrality of communities in health planning and decision making, yet three decades later, conceptualisations of rural communities as disempowered and distanced from urban centres of power continue [16,18,19]. Calls for meaningful multi-sectoral partnerships with communities recognise that collaboration is central to ensure acceptable, appropriate and effective responses to begin to tackle entrenched rural inequities [18]. Internationally, social, political and economic changes in rural environments, particularly associated with ‘mechanisation, modernisation and downsizing’ in agricultural industries [4] has impacted on rural social cohesiveness and contributed to the ‘circle of decline’ [7] being experienced in many rural locations.

Rebuilding or harnessing community capacity is integral to developing locally responsive health services [4] and is in the interest of communities and government as it draws together rural social capital, maximises the innate, adaptive, inventive and innovative nature of rural people [12,20] and leads to empowered communities capable of developing local solutions [21,22]. There are shared advantages for communities and government in terms of rural town survival, resilience, sustainability, and fiscal responsibility [23], but consistently, a lack of knowledge on how to build effective community/policy maker partnerships that empower communities and encourage citizen control and responsibility in local decision making is identified [4].

### The community participation agenda

**Figure 1:**
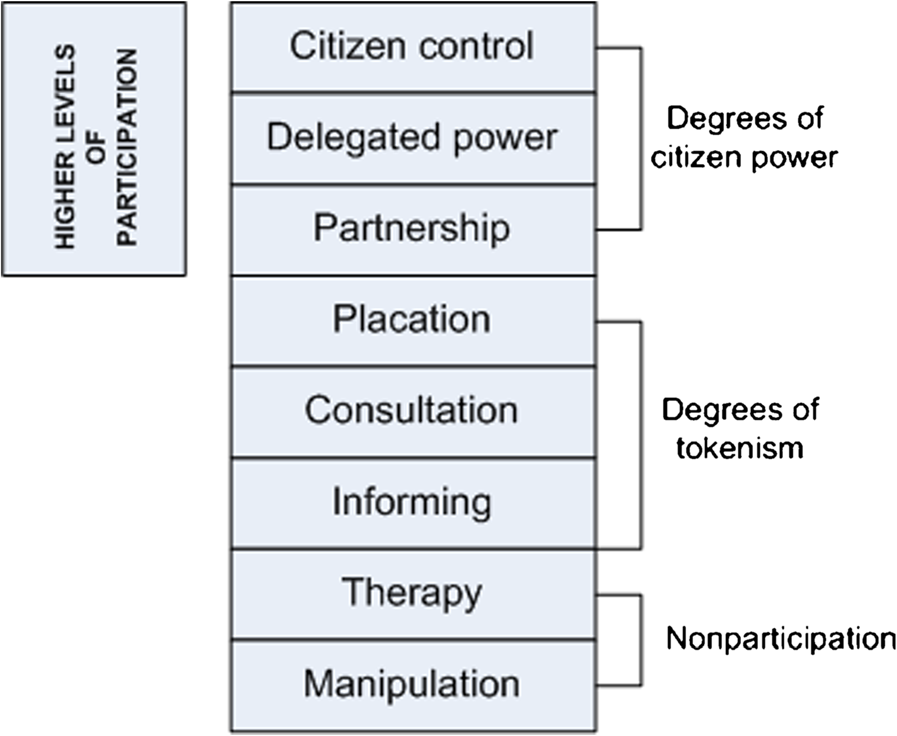
**Levels of participation.** Adapted from Arnstein [29].

Despite the desire to meaningfully engage communities in health care planning, and the adoption of community participation as central in the health agendas of many countries [4,6,7,11-13,24], researchers continue to debate models, approaches, motivations, definitions and operational challenges [22,25,26]. Most commonly, researchers define communities as groups bounded by geographic location [27], and participation as collective actions that harness socio-cultural affiliations, customs, values and beliefs through social interactions to influence and localise outcomes [28]. In theoretical terms, participation is understood to be multi-level, depicted as a ladder by Arnstein [29] (see Figure 1), or as a spectrum (see for example International Association for Public Participation [30]).

The seminal work of Arnstein [29] has been extensively cited and is influential in theories of participation and the interaction of power structures in society. Arnstein [29] argued that whilst participation is theoretically the cornerstone of democracy, in reality, large sections of the community are powerless and excluded from political and economic decision making. Participation is described in categorical terms as citizen power, and a typology proposed, illustrated by a ladder of participation, to highlight the divergence of views between those who have power and those who do not. She describes the ladder as an illustration of the different grades of participation, and by understanding these differences there can be greater understanding of citizens demands for meaningful, power redistribution and the tokenistic way in which participation is often considered by those in power [29].

At lower levels, participation is consultation or information provision, and at highest levels is full citizen control that involves the redistribution of power from ‘government to the governed’ [29]. While there is robust debate in the literature about Government agendas for community engagement [16,22], particularly from a neoliberal perspective [21] current policy in several countries [4,6,13,31] reflects a desire to engage communities at the higher level of Arnstein’s [29] ladder; partnership, delegated power and citizen control.

### The Australian rural context

As Australian rural researchers, our interest in Arnstein’s [29] higher levels of community participation is driven by the emphasis on community participation in the Australian healthcare reform agenda [11-13,31,32], international recognition of the lack of knowledge on how higher level community engagement is achieved [4], interest in the sustainability and empowerment of rural communities [4,12], and interest in policy agendas that promote local responsiveness [13]. Like many countries, Australian health care reform is driven by increased demand for health services, inequities in health care access and outcomes, issues of quality and safety, workforce mal-distribution and inefficiency and system fragmentation [13]. While Australia has a universal health care system, Medicare, there is recognition that a universal system does not result in universal access, with significant access and equity issues evident in rural areas [12,13]. The increasing emphasis on community participation, consumers, patients and citizens, to develop services that are locally tailored is evident in Australian policy [11,31] and the imperative for community participation to be central to decision making is mandated in National Safety and Quality Health Service Standards [33]. Key Australian reform documents state that policy, system and service reform must result in local responsiveness, flexibility and agility [11], and that ‘public voice and community engagement’ [13] is one of the most important levers to achieve a continuously improving health care system:

*Consumers should not only be the focus of the health system, they should be at the centre of decision-making in health. Both at a policy level and an individual level, consumer experiences and preferences should help lead health system reforms, alongside the evidence base. The reality of shared responsibility requires not just declaring it but building consumer health literacy and access to quality information and advice*[13].

While definitions of rural are debated [34], for the purposes of this article we refer to rural as areas outside capital cities and metropolitan centres. In Australia, community participation in rural areas is described as an important strategy to build self reliant and self determined communities, and in health policy terms, is viewed as central in developing locally, responsive healthcare that is based on rigorous population health needs assessment [10,31]. Researchers note the long tradition of rural community participation in Australian health services [35], that many communities demand involvement [22], and that the sustainability of rural health services is viewed as central to the sustainability of towns [36]. Kilpatrick [22] suggests, however, that there is a wealth of community participation in rural health service planning that is never reported and that given policy imperatives for higher level community engagement, there is an urgent need to capture examples and commit to ‘analysing the processes of community engagement in order to improve them’ [32]. There is a commitment to community participation but ‘reluctance by policy makers to analyse and measure’ [32] and at the practice level, little guidance on how policy is best enacted [4].

 Given international imperatives to develop locally responsive services and build sustainable empowered communities, research that investigates process and outcomes of community participation is of central importance for policy and practice. The aim of our study was to identify examples, in the international literature, of higher level community participation in rural healthcare.

## Methods

### Study design

We designed a scoping review to map the existing evidence base on higher level community participation in rural health. In progressing an agenda of exploration and analysis of the process of higher level community participation, our definition of higher level utilised Arnstein’s [29] categories of partnership, delegated power and citizen control, most commonly clustered as ‘citizen power’. Arksey and O’Malley’s [37] work on scoping reviews was useful in our conceptual thinking. Consistent with their work, we acknowledged that the first step was to ‘identify gaps in the evidence base’ and draw ‘conclusions from existing literature regarding the overall state of research activity’. Researchers have identified scoping reviews, as an effective means of capturing a range of literature on a topic [38] and for our purpose it was a useful approach to mapping and collating existing literature in a summary format that would be useful for policy makers and practitioners. Scoping reviews differ from systematic reviews, in that the focus is not on the assessment of quality as defined within a biomedical research paradigm [39], rather, the approach enables a broader range of literature to be captured, including all types of study designs [37]. Arksey and O’Malley [37] propose a methodological framework for scoping reviews to enable replication and strengthen methodological rigour. The five stages of their framework; identifying the research question, identifying relevant studies, study selection, charting the data, and collating, summarising and reporting results were utilised in this study.

### Identifying the research question

To guide the search strategy, and ensure that a broad range of literature was captured, the research question: ‘What examples of higher level community participation in rural healthcare exist in the international literature?’ was developed. In defining parameters it is recommended that wide definitions of key terms are initially adopted to ‘generate breadth of coverage’ [37] and we considered the broad terms appropriate for this stage.

### Identifying relevant studies

To balance the need for comprehensiveness with pragmatic cost and time limitations, we developed inclusion and exclusion criteria based on our review purpose (outlined in Table 1). A methodological limitation is that choices may have excluded relevant papers.

**Table 1:** Inclusion and exclusion criteria

**Criterion**	**Inclusion**	**Exclusion**
Time period	January 1990 and February 2012	Any study outside these dates
Language	English	Non-English
Type of article	Original research article published in a peer reviewed journal	Any article that was not original research and/or unpublished
Study focus	Community participation	No reference to community participation, i.e. individual consultation between health professional and client
Health service	Rural	No reference to rural health care services
Geographical place of study	International, developed countries	Developing countries
Population and sample	Mixed population sociodemographic	Reference to only a single sociodemographic factor i.e. gender, cultural group

 Key search terms were developed and a search of the Cochrane Library (see http://www.thecochranelibrary.com) identified one study on consumer consultation [40] and confirmed the absence of registered Cochrane reviews. The existing Cochrane review did not meet the inclusion criteria. A broad scan of Medline located a scoping review by Mitton et al. [41] who had scoped a similar topic, but not with a rural focus. Recognising that qualitative and mixed method studies can be difficult to locate, terms were mapped using SPIDER [42]. The phenomenon of interest was community participation. Linked descriptive terms were used to represent the types and levels of participation, to increase the range and depth of search results. Table 2 illustrates the search terms used noting that the term rural was used in all searches.

**Table 2:** Search terms

**SPIDER Tool**^**1**^	**Search terms**
**S**	(“rural” OR “regional”) AND (“population” OR “healthcare” OR “community”)
**P of I**	(“communit*” OR “consumer” OR “citizen”) AND (“participation” OR “engage*” OR
	“involve*” OR “partner*” OR “collaborat*” OR “develop*”)
	OR “cooperative behavio*” OR “stakeholder governance” OR “community network*” OR “community develop*” OR “social capital health services” OR “community-institutional relations” OR “community health planning” OR “health service*” OR “health planning”
**D/E/R**	“qualitative” OR “quantitative” OR “mixed method*” OR “community participation action” OR “case study” OR “cohort study” OR “quality assurance”

^1^ [S AND P of I] AND [(DER)].

The developed terms were used to search Medline, CINAHL, Proquest, Expanded Academic, Informit and Cochrane databases, with additional searches using Google Scholar.

### Study selection

Using the developed search terms 2467 articles were identified. An initial scan of title and abstracts identified large numbers of irrelevant studies, particularly those related to patient consultation and one off engagement activities that did not fit with Arnstein’s definition of higher level participation. Through a process of elimination, driven by inclusion/ exclusion criteria, 99 studies were identified as potentially relevant. Full text versions of the articles were obtained and, as a key parameter for our review was high level participation, each paper was reviewed by more than one team member for evidence of partnership, delegated power and citizen control. Discussion occurred between the researchers to ensure there was consensus on the level of participation identified.

Over one-third of publications found were from Australian rural health journals including the Australian Journal of Primary Health and the Australian Journal of Rural Health. Australian researchers published 40 of the 99 articles retrieved; the United States of America (USA) 16, Canada nine, United Kingdom (UK) five and New Zealand one. After review, 24 studies demonstrated Arnstein’s lower levels of participation [29], with publication dates between 1994 – 2011; 15 were Australian, seven from the USA, one from both the UK and New Zealand. Key topics covered by these 24 articles included consumer representation on health boards and governance, community consultation in strategic planning, strategies to involve community feedback in health care planning and design [43-45], and funding submission [46].

Overall, of the 99 articles located, innovative research methods for rural community participation were an emerging area, with eight articles published from 2006–2011; four were Canadian, with the remainder from USA, UK and Australia. Other topics covered were participatory action research design, development of theoretical frameworks or production of toolkits for consumer feedback and consultation [16], and development of conceptual frameworks for guiding or measuring processes [27]. The exploration of interagency partnerships [47-49] and workforce development [44,50,51] were considered by six articles. Conceptual discussion of community participation, defining key terms and highlighting issues for research and ethics were the focus of eight articles [52].

**Figure 2:**
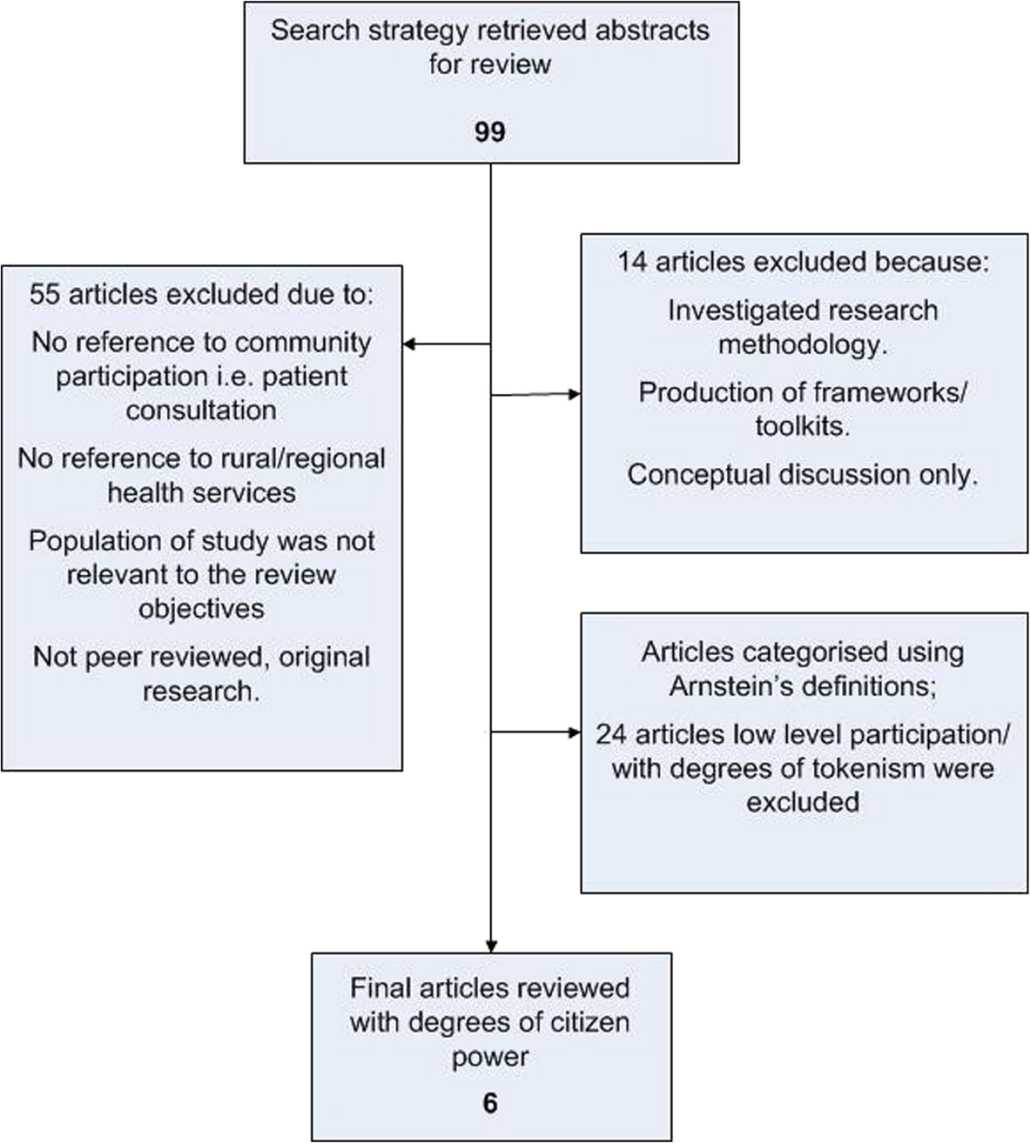
Process of article selection.

While the identified articles provided important background to our research question, the process of review for evidence of partnership, delegated power and citizen control only yielded six articles; published in the period 2003–2009. Three studies were conducted in rural USA [53-55], one in rural Canada [56] and two in rural Australia [57]. They included community capacity building [55,57], partnership development [35,55], and community involvement in health care design and development [35,53-57]. Figure 2 illustrates the process of study selection.

Consistent with the purpose of the review, to identify examples of high level participation that could be useful for policy and practice, we refined our final article selection to the six articles on rural health that most closely demonstrated higher level participation or Arnstein’s notion of ‘citizen power’.

### Data charting and collation

The fourth stage aligned with Arksey and O’Malley’s description of a charting approach. We developed summaries of each article and documented data related to author, year, location, study design, methods and sample (see Table 3).

**Table 3:** Articles with high-level community participation located in rural and regional health settings

**No.**	**Author**	**Year**	**Location**	**Intervention**	**Study design/methods/sample**
1	Broussard [53]	2003	USA	Development of community health networks	Case study with mixed methods survey; Two rural communities located in Louisiana, St Marys parish population 53 500, Vermillion parish population 50 755
2	Coady [56]	2009	Canada	Volunteers on community health boards	Qualitative study with focus groups; sample 45 volunteers, working on community health boards, population rural shire of 50 000
3	Johns [35]	2007	Australia	Health service redevelopment	Case study with individual and group interviews^1^; Two Tasmanian rural communities, greater Oatlands population 6101, Deloraine population 5524
4	Kegler [55]	2008	USA	Citizen involvement in paid/unpaid rural health leadership positions	Case study with mixed methods including postal survey, telephone interviews, and focus groups. California, sample N=243, 58% of respondents were from rural sites (n=140). Rural region/municipality combined area population 43 298
5	O'Meara [57]	2007	Australia	Council-led community capacity building	Qualitative design with content analysis of project documents and focus groups with community members; Gippsland, Victoria N= 9829; Korumburra 4465, Trafalgar 2685, postcode 3925 n= 2679 including Newhaven 428, San Remo 1017, Cape Woolamai 1234^2^
6	Huttlinger [54]	2004	USA	Primary healthcare community events	Case study with mixed methods survey; Rural Appalachia, Virginia area population N=1754^3^. Population N=3310 total health event participants, sample n=752 completed surveys, population including Wise, Virginia (3286) and Mountain City, Tennessee (2531) ^3^

^1^Number of interview participants not reported. ^2^Population numbers not reported in article, sourced from 2006 Australian census data. ^3^Population not reported in article, sourced from 2010 U.S census data.

### Summarising and reporting findings

Arksey and O’Malley describe the final stage of scoping reviews as an overview of the located studies. As the purpose of our study, was to identify examples of higher level community participation in rural healthcare, our reporting focuses on the six articles that best demonstrate this.

## Results and discussion

### The assessment of higher level community participation

The six studies had strong citizen investment, with power balanced in a mutually beneficial partnership [35,53-57]. Decision-making was democratic [35,53-57], with community members equipped and skilled in prioritisation, strategic business and financial planning. In the planning stages, stakeholders had a shared and agreed vision, control and responsibilities and leadership was shared and distributed [35,53-57]. In two studies, the idea for a partnership was initiated and driven by community leaders [35,54]. In all other studies, participation was initiated by government and driven by state initiatives [55] or funding through local council and research partnerships [53,56,57]. Coady [56] described that ‘the decentralisation of health decision making was welcomed as a meaningful opportunity to bring the voice of the community fully into the process of setting local health agenda’ [56]. Community representatives had bargaining influence over planning and outcomes and authority to veto or disagree with proposed plans and actions [35,53-57]. Researchers reported that shared power and leadership, transparency and accountability, enduring relationships, and mutual trust and respect, contributed to service outcomes [35,53,54,57]. In two studies, community members had the majority of decision making seats [35,57], however, in all studies power was retained by health professionals, paid coordinators, academics or health service managers as they held financial resources and ultimately decision-making powers [35,53-57].

Partnerships were needed to assess community strengths and resources, create management structures, facilitate comprehensive planning and negotiation, and to work through resistance [35,53,54,57]. However, gaining trust and acceptance was important and partners must be committed to long term outcomes for population health, and supportive of community ownership of health issues and solutions [35,57]. Utilising the knowledge, skills, resources and capacity building initiatives of universities and health services supported the achievement of outcomes [35,53,54,57].

Three of the studies reviewed [35,54,57] identified that higher level community participation is influenced by the nature of close knit rural communities and social interactions that support the development of new community sub-groups committed to local health care initiatives. Kegler [55] identified the importance of drawing on existing local leadership, and extending development opportunities to new leaders. In the rural context it was suggested that fewer resources provide a higher incentive for working together [35].

### Outcomes of higher level community participation

In the studies reviewed, outcomes from community participation were indicated, though not rigorously measured. Reported outcomes included awareness of the health services provided [35] and improved self efficacy, social capital and accountability [35,55,56]. Benefits to community members included learning new skills [35,53-57], particularly in strategic planning [53,56], meeting facilitation [35], grant submission [53] and leadership [55,56]. It was reported that paid and unpaid leadership positions were created [35,53-57], with benefits for the people employed and the broader community.

 It was reported that community participants enjoyed the learning process, the positive impact of contributing to healthcare in the community [56], new and strengthened relationships, reduced isolation, improved social support, and achieved a “strong sense of empowerment” [55]. Outcomes for the broader community included implementation of new public policy [56], new infrastructure and health services [35,54,57], and increased local employment positions [35,55]. Access to grant funding was described in some of the studies for community service development [53], and capacity building activities [35,57], with suggestions of small financial investment to reap large returns [55].

### Challenges in community partnerships

Some of the studies demonstrated that delegation of power to the community is challenging for some individuals or groups [56] and power may be shared conditionally and withdrawn in times of conflict [57]. In their study, O’Meara, Pendergast and Robinson described a situation of conflict where “council attempted to become more directive through the facilitators, rather than involving the community in defining their own solutions and strategies” [57]. Despite intentions of authorities to share power and ownership with the broader community, in all studies reviewed, the final decision-making powers were still held by a person or group in a professional, leadership position [35,53,54], such as local government [55-57]. Sustainability was supported by continuity of leadership [35,53,57], with one report of a study being temporarily suspended when a paid community facilitator was lost [57].

### Limitations of the studies

While all of the reviewed studies reflected elements of higher level participation, the study by Johns was closest to full citizen control [35], where power was only delegated to the partner health care organisation when managerial responsibilities exceeded the group’s capacity. In all of the studies, only scant details were provided about processes of nomination, election and representation with groups developed through self-selection or from existing leaders within the community [35,39,53-56]. In one study, participants included unemployed or low income volunteer community members [56] but in all studies little description was given about who was included or excluded and the rationale for these decisions. While one study described the community population as vulnerable and underserved [53], consideration of issues associated with working with marginalised populations was absent.

 None of the reviewed studies reported the use of web based interfaces or social media to mobilise and engage communities but instead relied on local media to disperse information, raise public perception and acceptance of community action and progress [53,54]. Early release of needs analysis research data in local newspapers and television news was identified as a cost effective method of gaining community interest but none considered the use of the internet to transfer or gather information.

The six studies had a similar study design, using qualitative research methods such as interviews and focus groups for collecting and analysing data on the participants’ experiences of participation [35,39,53-56]. This descriptive information provided an overview of possible outcomes for participants and the broader community, however no quantitative methods were used to measure or validate the outcomes reported.

## Conclusion

The limitations of this review related to size, breadth, inclusion and exclusion criteria, article selection and review are acknowledged. The very small number of articles identified is perhaps not surprising given contentions that a great deal of rural community participation is not reported. However, in an environment characterised by increasing interest in community participation in rural communities the need for rigorous research that explores and analyses higher level community participation is needed. Policy promotes community participation as highly desirable, but for many policy makers, practitioners and community members there are major gaps in understanding the purpose, process and outcomes.

## Abbreviations

WHO: World Health Organization; USA: United States of America; UK: United Kingdom.

## Competing interests

The authors declare that they have no competing interests.

## Authors’ contributions

VDS, AK, JF and PO conceived and designed the scoping review, and completed the first draft. JS developed the search strategy, completed the database searches and preliminary synthesis of findings; and provided input on early drafts. VDS and AK provided intellectual content to shape the findings and discussion. NH and JS made final decisions about article verification with consensus from all authors. NH collated all materials, completed the analysis of key findings and prepared the manuscript. AK edited the final manuscript, and all authors read and approved the final version prior to submission.

## Pre-publication history

The pre-publication history for this paper can be accessed here:

http://www.biomedcentral.com/1472-6963/13/64/prepub
